# Building peroxisomes: perspectives on plant peroxins

**DOI:** 10.1042/BST20230940

**Published:** 2026-09-18

**Authors:** Gabrielle C. Buck, Bonnie Bartel

**Affiliations:** Biosciences Department, Rice University, Houston, TX, U.S.A.

**Keywords:** peroxin, peroxisome biogenesis, peroxisome protein import, protein compartmentalization

## Abstract

Peroxisomes are dynamic organelles with diverse metabolic functions that are essential for life in plants and mammals. In humans, peroxisomal defects underlie peroxisome biogenesis disorders, which are often fatal. In plants, peroxisomes contribute to photorespiration and phytohormone production during plant development and are the sole site of fatty acid β-oxidation, which is critical for fat mobilization during germination. Despite their functional diversity, much of the machinery involved in building and maintaining peroxisomes is conserved across most eukaryotes. The model plant *Arabidopsis thaliana* is ideal for peroxisome studies due to its large peroxisomes, whole-organism and molecular assays for peroxisome function, and facile genetics. New applications of microscopy, computational modeling, and biochemistry have advanced the understanding of peroxisome biogenesis across eukaryotic life. These advances have generated insights into the functions of the proteins involved in peroxisome biogenesis, known as peroxins. Here, we review knowledge of *Arabidopsis thaliana* peroxins and discuss how new perspectives on peroxin functions across kingdoms inform future research on plant peroxisomes.

## Introduction

Peroxisomes are found in most eukaryotes and perform conserved and specialized metabolic functions. The most conserved peroxisomal reactions are fatty acid β-oxidation and reactive oxygen species metabolism [1–8]. Additional functions vary across species. Fungal peroxisomes are involved in pathogenicity, utilizing alternative carbon and nitrogen sources, and synthesizing specialized compounds [4]; mammalian peroxisomes play vital roles in development, antiviral immunity, and aging [4,7,8]; and plant peroxisomes are critical for development, stress response, and photosynthetic efficiency [5,6].

In plants and fungi, peroxisomes are the sole site of fatty acid β-oxidation, which in plants fuels embryogenesis, seedling growth, and stomatal opening [5,6]. Plant peroxisomes also help synthesize key growth and stress-signaling hormones, including auxin, jasmonates, and salicylate [5,6]. Moreover, peroxisomes house essential steps in C_2_ metabolism during photorespiration, converting toxic glycolate into glycine [9,10]. Thus, understanding peroxisome biogenesis and function will help illuminate plant development and adaptability, which underpins agricultural advancement.

Because peroxisomes import small and large molecules [11,12] and utilize simple, easily appended N- or C-terminal targeting signals [12–17], peroxisomes are attractive targets for engineering [12]. Furthermore, peroxisomal compartmentalization enables substrate concentration and reaction segregation. For example, targeting eight mevalonate pathway enzymes to peroxisomes while overexpressing several proteins involved in lumenal protein import dramatically increases geraniol production in yeast [18]. The recent rediscovery of peroxisome intralumenal vesicles [19] may provide additional opportunities for reaction partitioning. Modifications to crucial plant peroxisomal processes, such as photorespiration, β-oxidation, and the metabolism of reactive oxygen and nitrogen species, may provide avenues to improve crop germination, resilience, and yield [12,20]. Indeed, engineering a bacterial β-hydroxyaspartate cycle into Arabidopsis peroxisomes reduces carbon and nitrogen loss during photorespiration, providing a strategy to couple photorespiration to C_4_ metabolism [21]. Advancements in biotechnologies and products will be aided by understanding how peroxisome formation and dynamics affect metabolic flux.

**Figure 1:**
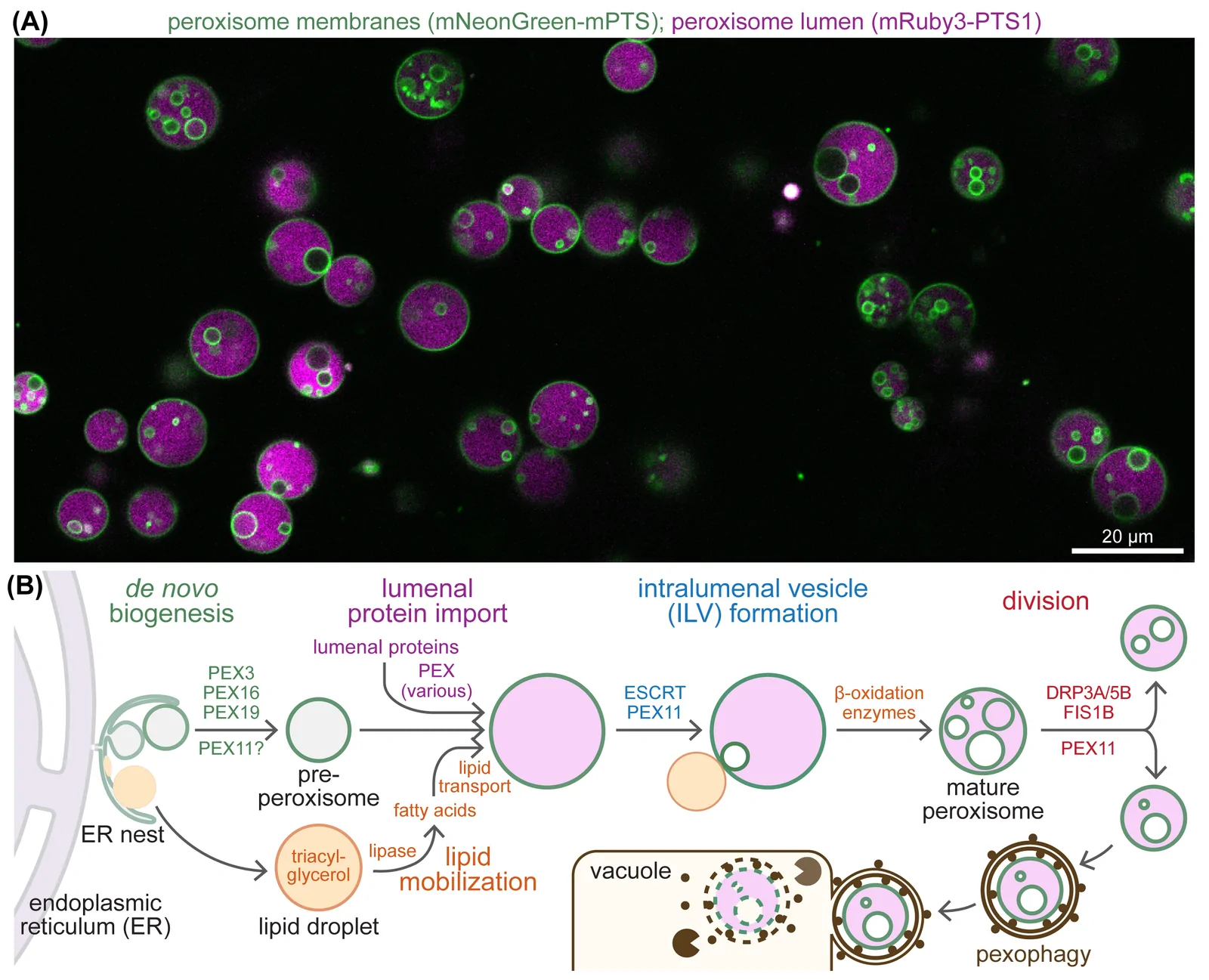
Plant peroxisome visualization and life cycle (**A**) Confocal micrograph of peroxisomes in cotyledon epidermal cells of a 3.5-day-old wild-type seedling carrying a reporter [19] that expresses mNeonGreen fused to a peroxisome membrane targeting signal (mPTS) from PEX26 (green) and mRUBY3-PTS1 marking the peroxisome lumen (magenta). (**B**) Plant peroxins (PEX proteins) assist in peroxisome biogenesis at the ER, lumenal protein import, intralumenal vesicle formation, and division, and presumably play roles in peroxisome degradation via specialized autophagy (pexophagy).

Although peroxisomes are often studied in yeast due to the ease of genetic manipulation, yeast are unicellular, limiting the assessment of peroxisomal contributions to multicellular life [22]. In addition to the well-known benefits of the reference plant *Arabidopsis thaliana* for genetics [23], Arabidopsis offers specific advantages for peroxisome research. Arabidopsis seedlings can have peroxisomes larger than an entire yeast cell [19,24–26], making Arabidopsis ideal for light microscopy of these organelles (Figure 1A). In addition, a suite of viable peroxisome-defective mutants coupled with whole-organism physiological and molecular assays allows straightforward monitoring of peroxisome function [5,27]. Finally, unlike mammals, intact Arabidopsis seedlings can be imaged via confocal microscopy, enabling exploration of peroxisome dynamics in living multicellular organisms. The similarities between plant and human peroxisomes suggest that characterizing peroxisome-related plant mutants may enhance our understanding of clinically relevant alleles [28–30].

Despite the functional diversity and metabolic plasticity of peroxisomes, many of the proteins involved in peroxisome biogenesis, called peroxins (PEX proteins), are conserved [31], and deciphering peroxin roles in one species can inform studies throughout eukaryotes. Although fungi [3,4] and protists [2] do not require peroxisomes for survival, most peroxins are essential for life in both humans [7,32,33] and plants [34–41]. Peroxins act throughout the life of the peroxisome—in biogenesis, lumenal protein import, division, and turnover (Figure 1B). In this review, we discuss the structure and function of plant peroxins, extend emerging lumenal protein import models from other organisms to plant peroxisomes, and highlight areas for future investigation. This synthesis has implications for understanding organellar evolution and advancing peroxisome engineering. Below, we discuss peroxins in functional groups (Table 1) responsible for inserting membrane proteins, targeting lumenal proteins, translocating receptor-cargo complexes into the organelle, recycling the lumenal protein-targeting machinery, and generating peroxisome membrane complexity.

**Table 1:** Arabidopsis peroxins

Peroxin	Function	Location
**Peroxisomal membrane protein (PMP) insertion**		
PEX16	PEX3 localization; PMP insertion	Membrane
PEX3A, PEX3B	receptor for PEX19; PMP insertion	Membrane
PEX19A, PEX19B	PMP chaperone, binds PEX3	Cytosol
**Lumenal protein receptors**		
PEX5	PTS1 receptor, binds PEX7	peroxisome-cytosol cycling
PEX7	PTS2 receptor, PEX5 dependent	peroxisome-cytosol cycling
**Docking/Pore**		
PEX13	lumenal protein import pore	peroxisome membrane
PEX14	binding of receptor-lumenal protein complexes	peroxisome membrane
PEX8	cargo unloading or chaperoning PEX5 to retrotranslocation pore	peroxisome lumen; peripheral membrane
**PEX5 ubiquitination**		
PEX4	ubiquitin-conjugating enzyme (E2)	cytosolic; tethered to peroxisome
PEX22	PEX4 tether	peroxisome membrane
PEX2, PEX10, PEX12	heterotrimeric RING type ubiquitin-protein ligases (E3); PEX5 retrotranslocation pore	peroxisome membrane
**PEX5 retrotranslocation**		
PEX1, PEX6	heterohexameric AAA ATPase	cytosolic; tethered to peroxisome
PEX26	PEX1/PEX6 tether	peroxisome membrane
**Peroxisome membrane shaping**		
PEX11A-E	peroxisome size control, intralumenal vesicle formation, proliferation	peroxisome membrane

## Insertion of peroxisome membrane proteins

Peroxisomes are born at specialized ER subdomains [42] termed ER nests [25] (Figure 1B). Peroxisome membrane proteins (PMPs) are targeted to peroxisomes through two pathways: either co-translational insertion into the ER followed by trafficking to nascent peroxisomes via ER nests or direct insertion into mature peroxisomes from the cytosol. For example, Arabidopsis PEX22 is ER-trafficked, whereas PEX26 appears to be inserted from the cytosol [19,25].

Recruitment and insertion of PMPs are performed by the early-acting peroxins—PEX3, PEX19, and PEX16. In Arabidopsis, two genes encode both PEX3 and PEX19 (Table 1). Plants expressing RNAi targeting one *PEX3* gene lack notable defects, whereas knocking down both *PEX3* genes confers elongated peroxisomes, suggesting redundancy [43]. Similarly, *pex19* single mutants lack peroxisome defects, but *pex19a pex19b* double mutants die during embryogenesis [44]. In contrast, Arabidopsis PEX16 is encoded by a single gene, and hypomorphic *pex16* mutants display physiological defects [45] and enlarged peroxisomes [39,43,45].

During peroxisome biogenesis, PEX16 in the ER membrane partitions to nascent pre-peroxisomes [46,47]. There, PEX16 recruits PEX3, another integral membrane protein [47,48], which in turn recruits ER-trafficked PMPs [49]. PEX16 may also recruit some PMPs directly [47]. Despite physiological defects, viable Arabidopsis *pex16* mutants lack notable alterations in the levels or membrane association of several PMPs [45]. Whether PMPs mislocalize to other membranes in Arabidopsis *pex16* mutants has not yet been reported.

PMPs that directly insert from the cytosol use PEX19 as a cytosolic receptor/chaperone [44,49–52]. Structural studies of yeast and human PEX19 reveal a disordered N-terminal domain (NTD) and a helical bundle C-terminal domain (CTD) that binds a PMP [53–56] via its membrane peroxisomal targeting signal (mPTS), typically a cluster of basic and hydrophobic residues and the adjacent transmembrane domain(s) [57–60]. Farnesylation of the Cys residue of the PEX19 C-terminal CaaX motif creates a hydrophobic pocket that increases PEX19-PMP affinity in yeast and humans [57,61,62]. Curiously, despite widespread conservation of the CaaX motif, PEX19 farnesylation is not essential for peroxisome biogenesis or function in plants, yeast, or mammals [44,63].

The PEX19-PMP complex is recruited by PEX3 in the peroxisome membrane. An amphipathic helix (AH) in the disordered NTD of PEX19 docks with a hydrophobic cleft in a region of the cytosolic domain of PEX3 distal to the peroxisome membrane [49,64–66]. PMP membrane insertion involves a weaker interaction between a second helix in the PEX19 NTD and a hydrophobic groove in PEX3 near the peroxisome membrane, activating the release of PMP cargo from PEX19 and preventing other cytosolic chaperones from binding PMPs [67]. PEX3 and PEX19 are also implicated in inserting membrane proteins into lipid droplets in human cells [68,69], which is intriguing given that both peroxisomes and lipid droplets can emerge from the same ER nest in Arabidopsis [25].

## Peroxisomal targeting signals and the receptor peroxins

**Figure 2:**
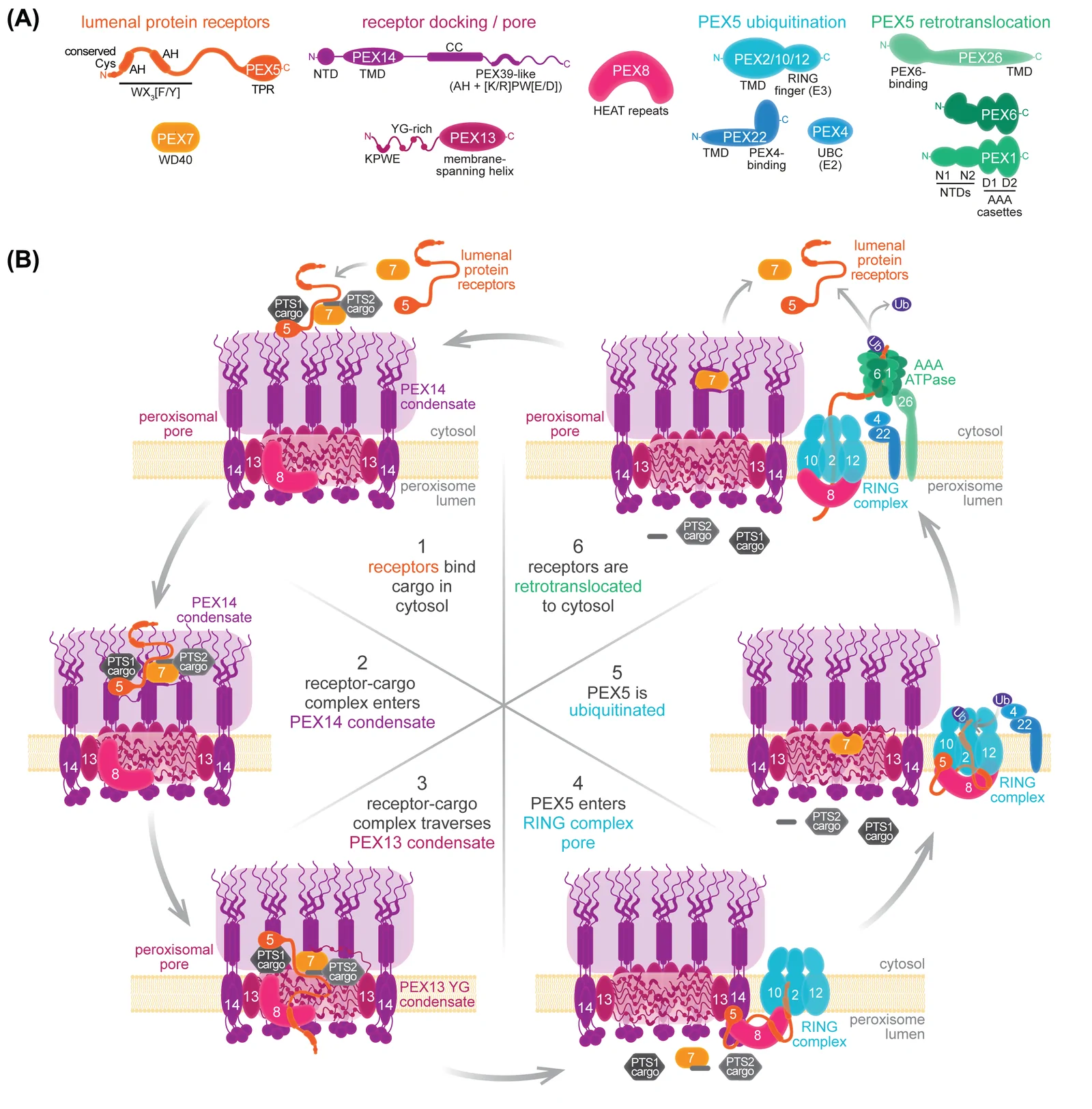
Peroxisome lumenal protein import (**A**) Domain arrangements of Arabidopsis peroxins (PEX proteins) involved in lumenal protein import. Proteins are not shown to scale. (**B**) Hypothetical model for import of PTS1 and PTS2 cargo proteins into the peroxisomal lumen in Arabidopsis based on conserved peroxin domains, interactions elucidated in Arabidopsis, and analogy to other systems. The PEX5 and PEX7 receptors dock with their cargo (1), enter the PEX14 condensate (2), and traverse the PEX13-condensate pore (3). After import, PEX5 enters the PEX2-PEX10-PEX12 channel, perhaps with assistance from PEX8 (4), is ubiquitinated at a conserved Cys residue by the RING peroxins using ubiquitin provided by PEX4 (5), and is retrotranslocated by the PEX1-PEX6 ATPase (6). PEX7 exits via the PEX13 channel (5) with the assistance of a PEX39-like motif in PEX14 (6).

Peroxisome lumenal protein import is mechanistically distinct from import into organelles that transit unfolded polypeptide chains across their membranes (e.g., ER, mitochondria, plastids). Instead, peroxisomes can import folded, even oligomeric, proteins [11] using a process resembling nuclear import [70]. Below, we discuss the peroxins executing this intriguing process (Figure 2).

Most peroxisomes use two convergent pathways to import lumenal proteins bearing a peroxisomal targeting signal (PTS) 1 or PTS2 (Figure 2B), although some invertebrates, including fruit flies and nematodes, have lost the PTS2 system [71,72]. The PTS1 is a tripeptide ([S/A][R/K][L/M]) at the C terminus [13–16], whereas the PTS2 is a nonapeptide (R[I/L]X_5_HL) in an α-helix near the N terminus [15,73–76]. The PTS1 is retained after import, whereas the PTS2 region is removed by the protease DEG15 in plants [77,78] or the related TYSND1 in mammals [79]; *Saccharomyces cerevisiae* lacks a DEG15 paralog [77], and yeast PTS2 proteins retain the signal after import [80].

In the cytosol, the PTS1 and PTS2 are recognized by the receptors PEX5 and PEX7, respectively (Table 1 and Figure 2B.1). The PTS1 binds to the PEX5 CTD composed of seven tetratricopeptide repeats (TPRs) [81–85], and the PTS2 binds to PEX7, a seven-propellered WD40 protein [84,86–92].

**Figure 3:**
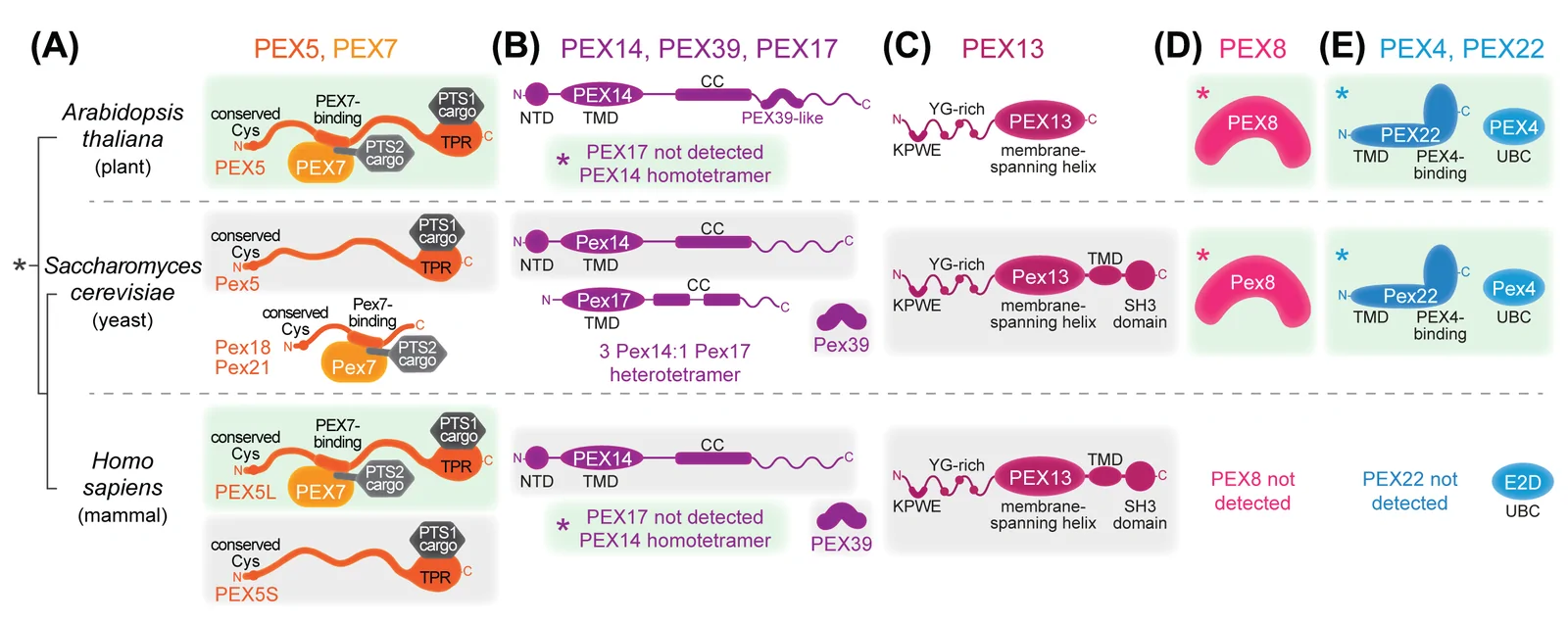
Several peroxins acting in lumenal protein import have adopted different domain arrangements during evolution Features conserved in two of three kingdoms are highlighted in green (plants and yeast or humans) or gray (yeast and humans), with asterisks marking the presumed forms found in the last common ancestor of plants, fungi, and animals. (**A**) Arabidopsis PEX5 (the PTS1 receptor) binds PEX7 (the PTS2 receptor) and resembles the long (L) human splice variant. Humans also express a short (S) splice variant without the PEX7-binding region. Yeast PTS2 import utilizes Pex7-binding Pex5 homologs that lack the TPR domain (Pex18 and Pex21 in *S. cerevisiae*). (**B**) Yeast Pex14 forms a heterotetramer with Pex17, whereas human PEX14 forms a homotetramer. Like humans, plants lack PEX17, implying that plant PEX14 forms homotetramers. A PEX39-like domain is inserted in the C-terminal region of Arabidopsis PEX14; PEX39 is a separate protein in yeast and humans. (**C**) Arabidopsis PEX13 lacks an SH3 domain and an additional transmembrane domain (TMD) found in the yeast and human PEX13 proteins. (**D**) PEX8 is found in plants and yeast, but not in mammals. (**E**) Plants and yeast tether the PEX4 ubiquitin-conjugating enzyme (UBC) to the peroxisome membrane via PEX22; humans use cytosolic UBCs in the E2D family to provide ubiquitin to the RING peroxins.

The landscape of interactions among the cargo receptors varies substantially across species (Figure 3A). For example, in mammals and plants, PTS2 cargo enters peroxisomes via PEX7–PEX5 interactions, whereas in fungi, Pex5-like co-receptors (e.g., Pex18, Pex21, and Pex20) accompany Pex7 and its cargo into the lumen [31,93–97]. Mammals [88,98–100] and monocots [101] express two splice variants of PEX5: a short isoform, PEX5S, that lacks the PEX7-binding region (similar to fungal Pex5) and a long isoform, PEX5L, that includes an alternative exon [87,88,100,101] in its N-terminal region with a PEX7-binding sequence similar to the yeast coreceptors [95,102,103]. Arabidopsis, on the other hand, expresses a single, full-length isoform of PEX5 that directly binds cargo-bound PEX7 [84,90,91] (Figure 3A). This obligatory coupling of PTS2 import to the PTS1 receptor in Arabidopsis provides a potential point of regulation. Interestingly, PTS1 cargo promotes PTS2 import in an *in vitro* system developed from pumpkin seedlings [104], and reducing PEX7 function in Arabidopsis impairs not only PTS2, but also PTS1 import [91], suggesting that the two import pathways are interdependent in plants.

In addition to binding PTS2 cargo and PEX5 (or a co-receptor), yeast and human PEX7 bind PEX39, a recently characterized cytosolic peroxin with a [K/R]PW[E/D] motif that binds the bottom face of the PEX7 propeller (opposite the PTS2-binding site) and an amphipathic helix (AH) that interacts with the top face and stabilizes the PEX7–PTS2 interaction [105]. PEX5 and PEX39 bind similarly to the PEX7–PTS2 complex, suggesting that before membrane translocation, the PEX39 AH is displaced by a helix in the unstructured N-terminal region of PEX5 (or a PEX5-like coreceptor), leaving PEX39 bound to PEX7 via the [K/R]PW[E/D] motif [103,105].

Curiously, plants appear to lack PEX39 (Figure 3B). However, a component of the peroxisomal import pore, PEX14 [106], contains a similarly spaced AH and KPWD motif in its unstructured CTD [103,105]. PEX14 is a PMP with a cytosolic C-terminus [107–110], and it will be interesting to learn whether PEX14 has co-opted PEX39 roles in plants.

## Translocation of lumenal proteins with their receptors into the organelle

The cargo-receptor complex enters the peroxisome via a pore formed by PEX13 and PEX14 [108,109,111] (Table 1 and Figure 2B). PEX13 oligomers form the core of the import pore [70,111,112]. Each PEX13 monomer adopts one of two orientations in the peroxisome membrane [111], and interactions among their N-terminal YG-rich domains form a phase-separated meshwork [111,112]. Although the membrane orientation of the PEX14 NTD may be dynamic [113], most evidence suggests that PEX14 spans the peroxisome membrane with its NTD in the lumen and oligomerizing coiled-coil regions in the cytosol [107–110]. Like Pex14, yeast Pex17 contains a transmembrane domain that traverses the peroxisome membrane and C-terminal coiled-coil domains [107,114,115]. In *S. cerevisiae*, three Pex14 proteins oligomerize with one Pex17, which stabilizes the Pex14 oligomer [107]. However, Pex17 has not been identified in plants [116] or mammals (Figure 3B), and in humans, the PEX14 coiled-coil domains intertwine as a homotetramer [109]. Even filamentous fungi have not maintained a canonical Pex17, but instead harbor both Pex14 and a protein resembling both Pex14 and Pex17 [117–119]. The absence of Pex17 beyond the fungal lineage, the rapid evolution of fungal Pex17, and the stoichiometries of the yeast and human PEX14 oligomers suggest that Pex17 is a recent fungal innovation (Figure 3B) or that plants and mammals both dispensed with PEX17.

Like the YG domains of PEX13, the coiled-coil and unstructured CTDs of yeast and human PEX14 oligomers undergo liquid–liquid phase separation [109,112]. Charged residues in the unstructured CTD contribute to PEX14 condensation and PEX5 recruitment [109]. Interestingly, the PEX14 and PEX13 condensates are immiscible [109], prompting a model in which PEX5 enters the PEX14 condensate on the cytosolic peroxisomal surface (Figure 2B.2) before traversing the membrane through the membrane-spanning PEX13 condensate (Figure 2B.3).

After passing through the PEX14 phase, WX_3_[F/Y] motifs in the unstructured PEX5 NTD interact with the PEX13 YG meshwork, carrying the PEX5-bound cargo through the pore [111]. Also in the PEX13 meshwork, the remaining PEX39 [K/R]PW[E/D] interaction with PEX7 is displaced by a conserved KPWE motif in the YG-rich N-terminal region of PEX13 [103,105]. In the lumen, conserved FL motifs in the PEX14 NTD interact with PEX5 WX_3_[F/Y] motifs [120–125], consistent with a reported PEX5 binding site in the N-terminal region of Arabidopsis PEX14 [84]. This strong binding could impede PEX5 diffusion back to the cytosol [108]. Additional import directionality may be provided by the subsequent binding of PEX5 to a complex of three Really Interesting New Gene (RING) finger-containing ubiquitin-protein ligases (PEX2, PEX10, and PEX12) that acts in PEX5 recycling and/or binding to PEX8, the only known fully lumenal peroxin [41,126–132].

## PEX8

Until its recent discovery in plants [41,133], PEX8 was thought to be restricted to fungi; PEX8 has not been reported in animals (Figure 3D). Because yeast Pex8 binds Pex5 [126–132], Pex13 [134], and the Pex2-Pex10-Pex12 RING complex [128,134,135], Pex8 may chaperone Pex5 from the import pore to the RING complex and/or help dissociate PTS cargo from the receptors (Figure 2B.4).

**Figure 4:**
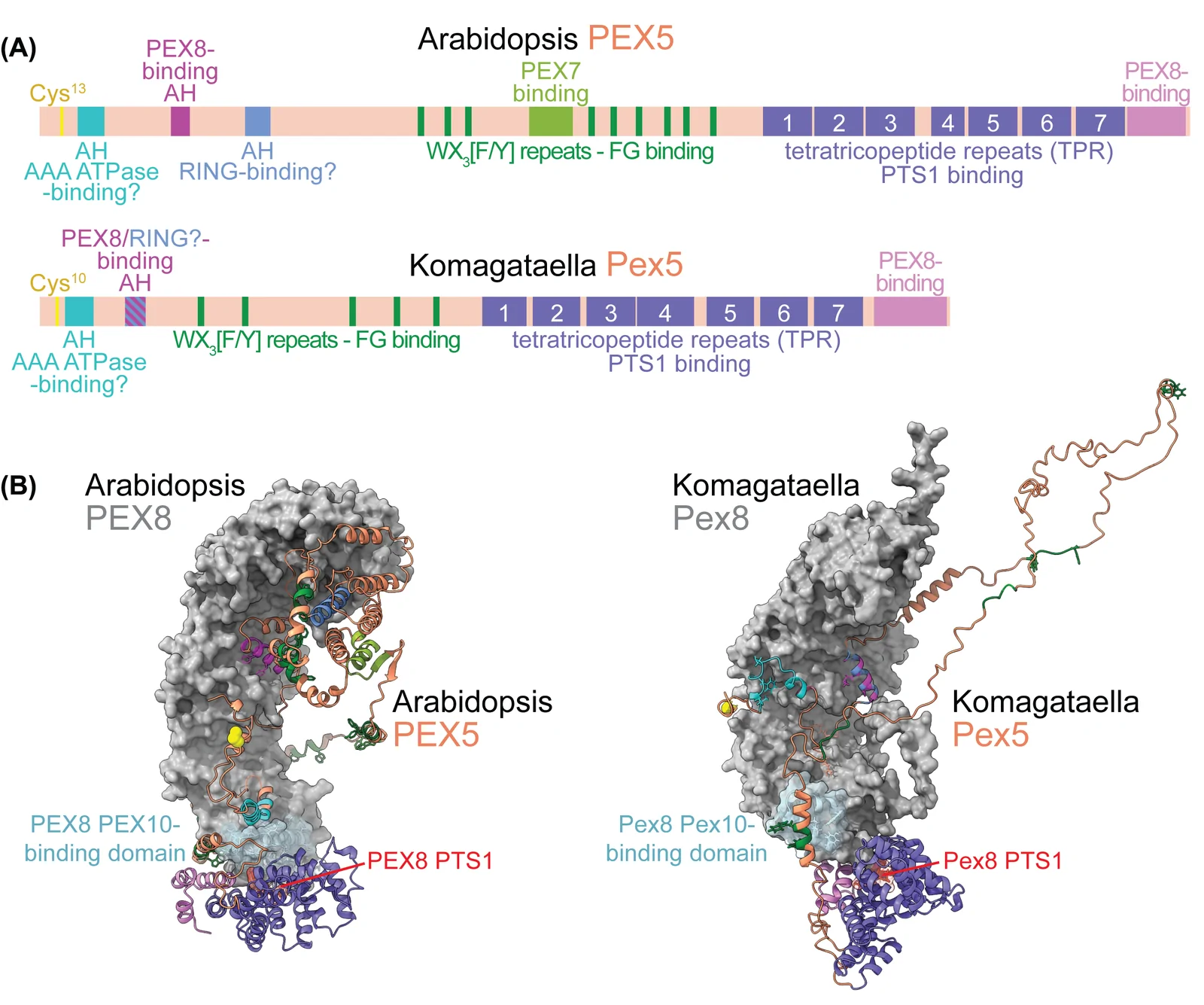
Predicted interactions of Arabidopsis PEX5 and PEX8 (**A**) Domain structure of Arabidopsis (top) and Komagataella (bottom) PEX5, indicating the positions of predicted amphipathic helices (AH) that may facilitate binding with other peroxin partners. (**B**) The AlphaFold-predicted structure of Arabidopsis PEX8 (gray, space fill) in complex with Arabidopsis PEX5 (depicted as a ribbon colored as shown in panel A, left) resembles the *K. pastoris* Pex8-Pex5 complex structure (right) [132]. Critical residues of WX_3_[F/Y] motifs are shown as sticks, and the conserved cysteine is shown as yellow spheres. Residues from some notable PEX8 domains (PTS1, red; PEX10-binding domain, light blue) are shown as sticks behind a translucent surface.

PEX8 is a solenoidal Huntingtin, EF3, a-PP2A, TOR1 (HEAT repeat) protein (Figure 4B) [41,132,133], like several nucleoporins (NUPs) [136] and karyopherins [137,138] involved in nuclear protein import. The superhelical scaffolds, Nup192 (NUP205 in vertebrates and plants) and Nup188, interact with FG repeats and are highly flexible, possibly aiding pore assembly and elasticity (to adjust to cargo size) [136,139]. Similarly, HEAT-repeat karyopherins (e.g., importin-β) facilitate nuclear import by binding receptor-cargo complexes [138,140,141], interacting with the FG motifs [137,142], and regulating phase separation in the nuclear pore meshwork [143,144]. Because Arabidopsis PEX8 localizes to peroxisomal puncta [41], it will be interesting to learn whether PEX8 partitions within the pore hydrogel to play similar roles in peroxisome protein import (Figure 2B.3).

PEX8 often appears to contain both a PTS1 and a PTS2. However, in several species, including *S. cerevisiae* and *A. thaliana*, the PTS2 is located at a non-canonical position near the center of the protein [41,126]. Curiously, neither the PTS1 nor the PTS2 is required for PEX8 peroxisomal localization or import function [41]. As expected, the TPR domain of Pex5 from *Komagataella pastoris* (formerly *Pichia pastoris*) binds the Pex8 PTS1 (Figure 4B); however, the association is weaker than that of Pex5 with other PTS1 cargo [132]. Instead, Pex5-Pex8 binding is primarily driven by the association of Pex8 with a bundle of three stabilized helices in the unstructured Pex5 NTD [132]. One of these helices, H3, is visible in cryo-EM analysis of the *K. pastoris* Pex5-Pex8 complex [132], and the homologous Pex5 helix in *S. cerevisiae* contributes to PTS1 import efficiency and binds Pex8 in AlphaFold models [135]. This helix was proposed to correspond to an amphipathic helix, AH2, in vertebrate PEX5 that associates with the RING complex [108]. However, in structural predictions of the Arabidopsis PEX5–PEX8 complex [41], the predicted plant AH2 [108] does not appear to bind PEX8. Instead, plant PEX5 appears to bind PEX8 via a third predicted helix (Figure 4) with Phe residues at the positions of the Met and Phe residues of H3 that are critical for Pex5*–*Pex8 binding in fungi [132]. Vertebrate PEX5 also contains a third (and fourth) predicted AH that is not necessary for peroxisomal protein import [108], unlike the fungal H3 [132]. These differences are intriguing because PEX8 is conserved in plants and fungi but is apparently absent from vertebrates (Figure 3D), implying that vertebrate PEX5 does not require PEX8 to release PTS cargo or to associate with the RING complex. It will be interesting to learn whether the vertebrate peroxisomal pore and RING complex associate via another mechanism, or whether the metazoan PEX8 is sufficiently divergent to have evaded detection by *in silico* methods.

## Receptor retrotranslocation

In fungi and vertebrates, PEX5 is ubiquitinated on a conserved Cys residue near the N-terminus by the PEX2–PEX10–PEX12 RING complex [145–147]. This ubiquitination enables retrotranslocation of PEX5 back to the cytosol and contributes to import directionality (Figure 2B.5). In fungal peroxisomes, PEX8 chaperones PEX5 while interacting via its concave face with the RING complex (Figure 2B.5), primarily through hydrophobic interactions with a lumenal PEX10 loop as the PEX5 N-terminus enters the RING complex pore [135]. The second amphipathic helix (AH) in the *Xenopus laevis* PEX5 N-terminal region interacts with the RING complex and may help orient the N terminus for insertion and position the conserved Cys residue for ubiquitination by the cytosol-facing RING domains [108].

In fungi, a peroxisome-tethered ubiquitin-conjugating enzyme, Pex4, provides ubiquitin to the RING complex [145,146]. Similarly, Arabidopsis PEX4 has ubiquitin-conjugating activity [148,149] and is tethered to the peroxisome membrane by PEX22 [149,150]. Mammals lack PEX4 and PEX22 [31]; instead, three cytosolic E2D-family enzymes provide ubiquitin-conjugating activity [151] (Figure 3E).

Following mono-ubiquitination, PEX5 is retrotranslocated by an ATPase associated with diverse cellular activities (AAA) complex consisting of a heterohexamer of alternating PEX1 and PEX6 monomers [40,152–158], which is tethered at the peroxisome membrane by PEX26 (Pex15 in yeast) [156,159–161] (Figure 2B.6). In vertebrates, the PEX5 AH1 is required for the ATPase to pull PEX5 out of the membrane [108,157]. Retrotranslocation-induced unfolding of PEX5 during export through the RING complex pore presumably releases PEX5 cargo in the lumen [108,157,162] (Figure 2B).

Although PEX5 ubiquitination has not been directly demonstrated in plants, the PEX5 Cys residue that is ubiquitinated in other systems [163–165] is conserved in Arabidopsis PEX5 [166] (Figure 4A), the Arabidopsis RING peroxins have *in vitro* ubiquitin-protein ligase activity [167], and PEX5 recycling is impaired when PEX4 is mutated [168], suggesting that PEX5 recycling proceeds similarly in plants. In addition, PEX5 levels are elevated in Arabidopsis RING peroxin mutants [169] or following proteasomal inhibition [168] and reduced in several *pex6* and *pex26* mutants [40,152,156,170]. Moreover, mutations in RING or PEX4 peroxins restore PEX5 levels in *pex6* and *pex26* mutants [156,171,172]. Together, these data imply that PEX5 marooned in the retrotranslocation channel is polyubiquitinated and degraded, a process that can be countered by reducing PEX5 ubiquitination.

As PEX5 is unfolded and returned to the cytosol, bound PEX7 would presumably be released along with its PTS2 cargo into the lumen (Figure 2B). In vertebrates and yeast, PEX7 returns to the cytosol the way it entered—by the binding and hand-off between [K/R]PW[D/E] motifs from PEX13 to PEX39, which extracts PEX7 from the pore hydrogel [103]. This PEX39 function may be performed by the PEX39-like region in the cytosolic CTD of PEX14 in plants (Figures 2B and 3B).

## Peroxisome membrane dynamics and PEX11

In addition to *de novo* peroxisome biogenesis, peroxisomes are reported to divide (Figure 1B), although this process has not been directly observed in plants. Division occurs through three stages: elongation, constriction, and fission [173]. The only peroxisome-specific protein implicated in this process is PEX11, a membrane peroxin with multiple isoforms in all examined peroxisome-containing eukaryotes [31]. In Arabidopsis, the five genes encoding PEX11 fall into three clades—PEX11A, PEX11B, and PEX11C/D/E—that differently impact peroxisome morphology and/or abundance when overexpressed [174,175] or mutated in various combinations [26,176]. Similarly, PEX11 isoforms from fungi and mammals promote peroxisome expansion or tubulation [177]. The constriction mechanism of peroxisome division is unknown, but fission is orchestrated by a set of dynamin-related proteins (DRPs) recruited to the membrane by FISSION1. The study of peroxisome division is complicated by multiple PEX11 isoforms and the involvement of DRPs and FISSION1 in the division of other organelles [173].

PEX11 has roles in peroxisome membrane dynamics beyond peroxisome division. For example, Arabidopsis PEX11 is involved in intralumenal vesicle (ILV) formation [26]. Peroxisomal ILVs (Figure 1A) were recently rediscovered and may aid in peroxisomal catabolism of insoluble fatty acids by serving as scaffolds that hold lipids within the vesicle bilayers [19]. In addition to keeping fatty acids accessible to β-oxidation enzymes, ILV formation counters the peroxisome expansion that accompanies lipid mobilization. As with multivesicular endosomes, peroxisomal ILV formation involves the endosomal sorting complexes required for transport (ESCRT) machinery [19]. Moreover, the plant-specific early ESCRT component FREE1 interacts with PEX11E [178], supporting the role of PEX11 in ILV formation. However, FREE1 does not interact with PEX11A or PEX11B [178], hinting at divergence between these isoforms.

Indeed, the five PEX11 isoforms have distinct roles in ILV formation. Double mutants in the PEX11C/D/E clade lack obvious defects, suggesting redundancy, whereas *pex11a* and *pex11b* show slight defects that are exacerbated in the double mutant [26]. In seedling cotyledons, both *pex11a pex11b* double mutants (*pex11ab*) and *pex11c pex11d pex11e* triple mutants (*pex11cde*) have enlarged peroxisomes with sparse ILVs. However, *pex11ab* peroxisomes shrink and form some ILVs as lipid stores decline, require lipid mobilization to drive aberrant peroxisome enlargement, and mobilize lipids normally [26]. In contrast, *pex11cde* exhibits whole-organism developmental defects, large peroxisomes with negligible ILVs in multiple tissues, accumulation of the membrane lipid phosphatidylcholine, and lipid droplet retention, indicating that this clade is necessary for normal lipid mobilization [26]. Thus, PEX11A and PEX11B are needed for ILV formation during rapid lipid mobilization in early seedling development, whereas PEX11C/D/E control homeostatic ILV formation throughout development.

Although the different PEX11 clades have distinct functions, seedlings survive with at least one PEX11 isoform [26,176]. However, the *pex11* quintuple mutant is seedling lethal [26,176]. Most other higher-order and non-redundant peroxin null mutants display embryonic or gametophytic lethality [34–41,44], indicating that PEX11 function is not critical until after embryonic development. Indeed, *pex11* quintuple mutant seedlings have even more peroxisomes than wild-type seedlings [26], demonstrating that PEX11 is not essential for peroxisome biogenesis. This seedling lethality indicates an essential role for PEX11 in peroxisome function (beyond biogenesis), perhaps in shaping ILVs that enable lipid utilization following germination.

## Remaining mysteries in peroxisomal protein import and biogenesis

Although our understanding of peroxisome biogenesis and protein import has trailed that of better-studied organelles, recent discoveries mark a renaissance in peroxisome research. Computational advances [179] provide new perspectives on existing datasets and opportunities to leverage bioinformatic strategies to generate and quickly evaluate hypotheses. New multi-omic datasets and structural prediction and analysis programs, such as HHPred [180,181], AlphaFold [182–184], and Foldseek [185], have revealed new peroxin genes [105] and peroxin orthologs in new species [41,133,186]. By exploiting these advances, it will be fascinating to uncover the full extent of peroxin conservation and any peroxins remaining to be discovered.

Despite functional conservation, identifying peroxin orthologs across species is complicated by limited primary sequence conservation, especially among the non-enzymatic peroxins, coupled with evolutionary shuffling of peroxin functions among different proteins (Figure 3). A few peroxins are missing from some kingdoms. PEX8 and PEX22 are found in fungi and plants but not in animals (Figure 3D,E), and PEX14 appears to fulfill the role of fungal Pex17 in plants and mammals, which lack Pex17 homologs [116] (Figure 3B). Some import roles are performed by different proteins in different kingdoms. For example, the Pex7-binding function in the NTD of PEX5/PEX5L in plants [90,187] and mammals [88,98–100,102,103] is co-opted by the yeast PEX5-like coreceptors (Figure 3A). Moreover, plant PEX14 harbors a domain similar to the PEX7-binding region in fungal and mammalian PEX39 [103,105] (Figure 3B). Interestingly, the CTD of PEX13 in mammals and yeast contains a transmembrane domain and a Src homology 3 (SH3) protein-interaction domain [188] that are absent from plant PEX13 [38,189] (Figure 3C). As plants have notably fewer SH3-domain proteins than animals or yeast [190], this domain on PEX13 may be a relatively recent invention. Alternatively, a membrane-anchored peroxin that functions as the opisthokont PEX13 SH3 domain may yet be discovered in plants. Investigation into these missing functions and domain swaps will inform the evolutionary origins and trajectories of peroxisomal complexes.

Advances in microscopy have revealed peroxisome morphology and dynamics in greater detail, illuminating peroxisome biogenesis and organelle interactions. Imaging the unusually large peroxisomes in germinating Arabidopsis seedlings led to the rediscovery of peroxisomal ILVs [19] (Figure 1A), which were noted in early micrographs of plant peroxisomes [43,191–194], challenging the common view of peroxisomes as simple, single-membrane organelles. Although the ER has long been implicated as the site of *de novo* peroxisome biogenesis, live-cell imaging of Arabidopsis seedlings identified specialized ER subdomains, ER nests, as the site of concurrent peroxisome and lipid droplet biogenesis [25]. The discoveries of ILVs and ER nests imply complexities in protein sorting, particularly of PMPs, that remain to be elucidated.

Many of the intricacies governing the peroxisome lifecycle (Figure 1B) remain enigmatic. The trafficking of the early-acting peroxins (PEX3, PEX19, and PEX16), their relationship to ER nests, and their roles in the insertion and sorting of various PMPs require clarification. Differential sorting of lumenal and membrane peroxisomal proteins will be fascinating to study; proteins sorting exclusively to ILVs or to a subset of ILVs may provide insight into sub-organellar organization and how peroxisomes coordinate their metabolism. Furthermore, some ILVs appear to contain PTS1 proteins [19] (Figure 1A); might peroxisomal lumenal proteins traverse both outer and inner membranes simultaneously, as in nuclear import? The end of the peroxisome lifecycle, pexophagy, is also poorly understood, particularly in plants, where peroxisomal targets for the autophagic machinery have not been identified [195]. Although several Arabidopsis peroxins harbor apparent binding motifs for ATG8 [196], which docks the autophagy machinery [197], whether these proteins influence pexophagy *in vivo* remains to be determined. Instead of destroying the entire organelle, the peroxins involved in PEX5 retrotranslocation might also help remove PMPs or lumenal proteins for ubiquitin-dependent degradation [198–200]. It will be interesting to learn the full complement of proteins that control ER nests, ILVs, and peroxisomal proteostasis, perhaps by examining these structures and processes in various peroxin mutants.

As the similarities between nuclear and peroxisomal protein import become increasingly apparent, it is tempting to speculate that these processes are evolutionarily related. Both nuclei and peroxisomes are ER-derived, can import fully folded proteins, utilize phase-separated FG- or YG-rich intrinsically disordered regions of pore proteins as selectivity barriers [70,109,111,112,136,201], and employ flexible cytosolic receptors that sometimes use adaptors to bind different targeting signals [70,138,202]. The receptors interact with the FG/YG pore domain and bind to lumenal domains with high affinity, enabling directional import [108,111,141]. Some receptors also return to the cytosol and/or facilitate export through the pore [103,141]. Because nuclear protein import is well studied, these similarities may aid in deciphering peroxisomal protein import and in uncovering the roles of poorly understood peroxins (such as PEX8). A fuller understanding of peroxisome protein import will provide new insights into the evolution of organelles and the eukaryotic cell.

Although conceptually similar, nuclei utilize dramatically more proteins than peroxisomes to accomplish import, and there is not a one-to-one correspondence between nuclear and peroxisomal import components. PEX14 is particularly multifunctional compared with the nuclear pore complex, with both a flexible, cytosolic filament that docks PEX5 at the pore and a lumenal NTD with high affinity for PEX5 [120–125] that may help enforce pore directionality [108]. In contrast, receptors bind the cytosolic and lumenal faces of the nuclear pore using separate filament and FG (WX_3_[F/Y]-binding) nuclear basket proteins, the latter of which also aids in cargo dissociation [141]. Given the various roles of PEX14, it is notable that PEX14 is the only peroxin known to be dispensable for plant viability, and peroxisomes in *pex14* null mutants still import some lumenal proteins [106].

Despite the recent renaissance in peroxisome research, many mechanisms underlying peroxisome biogenesis remain mysterious. However, new tools, researchers, and community-building efforts suggest an exciting future for the field. This resurgence, coupled with the expansion of synthetic biology and organelle engineering, will lead to discoveries that advance human health, pharmaceutical and natural product manufacturing, and agricultural resilience and efficiency.

## Perspectives

- Peroxisomes are metabolic hubs built to sequester, withstand, and inactivate oxidative byproducts produced during both conserved and specialized metabolism. In plants, peroxisomes are essential, aiding in seedling development and stress responses.
- Despite their discovery 70 years ago, the biogenesis of peroxisomes is poorly understood. Peroxisomes are often described as simple organelles, but emerging evidence reveals additional complexity in the formation of this adaptive organelle.
- Although research on peroxisomes is limited compared with other organelles such as mitochondria or chloroplasts, recent studies highlighting intriguing similarities between the import of folded proteins into peroxisomes and into the nucleus have provided new mechanistic models and opened avenues for future exploration.

## Open Access

Open access for this article is provided via our Subscribe to Open programme, made possible through the support of our subscribing institutions. Learn more here: https://portlandpress.com/pages/open_access_options_and_prices#conditional.

## Abbreviations

AAA: ATPase associated with diverse cellular activities
AH: amphipathic helix
CTD: C-terminal domain
DRP: dynamin-related protein
ER: endoplasmic reticulum
ESCRT: endosomal sorting complexes required for transport
HEAT: Huntingtin-EF3-a-PP2A-TOR1
ILV: intralumenal vesicle
mPTS: membrane peroxisome-targeting signal
NTD: N-terminal domain
NUP: nucleoporin
PEX: peroxin
PMP: peroxisome membrane protein
PTS: peroxisomal targeting signal
RING: really interesting new gene
SH3: Src homology 3
TPR: tetratricopeptide repeat
